# Decrease of miR-202-3p Expression, a Novel Tumor Suppressor, in Gastric Cancer

**DOI:** 10.1371/journal.pone.0069756

**Published:** 2013-07-25

**Authors:** Yu Zhao, Chenglong Li, Ming Wang, Liping Su, Ying Qu, Jianfang Li, Beiqin Yu, Min Yan, Yingyan Yu, Bingya Liu, Zhenggang Zhu

**Affiliations:** Shanghai Key Laboratory of Gastric Neoplasms, Department of Surgery, Shanghai Institute of Digestive Surgery, Ruijin Hospital, School of Medicine, Shanghai Jiao Tong University, Shanghai, China; Queen Elizabeth Hospital, Hong Kong

## Abstract

Emerging studies have indicated that microRNAs are involved in the development and progression of cancer. Here we found that miR-202-3p was frequently down-regulated in gastric cancer tissues. Overexpression of miR-202-3p in gastric cancer cells MKN-28 and BGC-823, markedly suppressed cell proliferation and induced cell apoptosis both *in vitro* and *in vivo*. Furthermore, Gli1 expression was frequently positive in gastric cancer tissues and inversely correlated with miR-133b expression. We demonstrate that the transcriptional factor Gli1 was a target of miR-202-3p and plays an essential role as a mediator of the biological effects of miR-202-3p in gastric cancer. MiR-202-3p also inhibited the expression of γ-catenin and BCL-2. Taken together, these findings suggest that miR-202-3p may function as a novel tumor suppressor in gastric cancer and its anti-tumor activity may attribute the direct targeting and inhibition of Gli1.

## Introduction

Gastric cancer (GC) is one of the most common malignancies and the second most common causes of cancer related death worldwide [1]. Although advances in treatment, the survival rate of patients with GC is disappointing, this is mainly due to the paucity of specific therapeutic targets. Therefore, it is crucial to identify the molecular mechanisms underlying the development of gastric cancer.

MicroRNAs (miRNAs) are a class of small (18–25 nucleotides), noncoding, single-stranded endogenous RNAs. They suppress protein expression by interacting with perfect or imperfect complementary sequences located in the 3′untranslated regions (3′UTRs) of target genes. Studies over the past few years have shown that dysregulated miRNAs play important roles in various cancers [1]–[4], including GC [5]–[13]. Data from study on miRNAs and their target genes may provide exciting therapeutic opportunities [3].

We have recently identified several miRNAs deregulated in GC, such as downregulation of miR-126 [14], miR-409-3p [15], miR-625 [16], and upregulation of miR-21 [17], miR-301a [18]. Although many miRNAs have been identified associating with GC, the mechanism of these miRNAs in gastric tumorigenesis still needs to be investigated. In our previous work, numerous putative miRNAs that showed differential expression between GC tissues and their adjacent non-tumor tissues from 28 patients were identified [19]. Among them, miR-202-3p was one of the most significantly decreased miRNAs. However the role of miR-202-3p in GC is rarely investigated.

MiR-202-3p, previous named hsa-miR-202, located within a chromosomal fragile site in 10q26. Deletion of the fragile site has been associated with endometrial [20], brain tumors [21], [22]. MiR-202 has been reported to be deregulated in breast cancer [23], [24], cervical squamous cell [25], colorectal cancer [26] and follicular lymphoma [27]. Petrocca et al. found that miR-202 was down-regulated in chronic gastritis [28]. MiR-202 has been shown to be down-regulated by 4-hydroxynoneal (HNE) in HL-60 leukemia cells [29]. A recent study also showed that miR-202 directly targets proto-oncogene MYCN, resulting in the inhibition of neuroblastoma cell proliferation [30].

Here, we demonstrate a general decrease in miR-202-3p expression level in 150 GC tissues compared with the non-tumor tissues and find that the miR-202-3p levels are associated with tumor size and patients age. Besides, we discovered, for the first time, that miR-202-3p could inhibit the growth and induce apoptosis of GC cells both *in vitro* and *in vivo* by directly targeting the transcription factor Gli1 and inhibiting expression of Gli1 target genes γ-catenin and BCL-2.

## Materials and Methods

### Ethics Statement

Written informed consent has been obtained from all participants. The study was approved by the Human Research Ethics Committee of Ruijin Hospital, School of Medicine, Shanghai Jiao Tong University (HREC 08-028), the Laboratory Animal Ethics Committee of RuiJin Hospital (LAEC 11-062). Animal procedures were carried out according to a protocol approved by the Institutional Animal Care and Use Committee (IACUC) at Shanghai Jiao Tong University, Shanghai, China.

### Cell Culture

Human GC cell lines SGC-7901 and BGC-823 were purchased from Shanghai Institutes for Biological Sciences, Chinese Academy of Sciences (Shanghai, China). MKN-45 and MKN-28 cell lines were obtained from the Japanese Cancer Research Resources Bank (Tokyo, Japan). NCI-N87, AGS, KATO III and SNU-1 cell lines were originally purchased from the American Type Culture Collection (Manassas, VA, USA). Human embryonic kidney cell line 293T (HEK-293T) was preserved in our institute. Cells were stored, recovered from cryopreservation in liquid nitrogen and used at early passages. All cells were maintained in RPMI-1640 medium plus 10% fetal bovine serum (FBS) and cultured in 5% CO2 humidified atmosphere. Exponentially growing cells were used for experiments.

### Patient Tissues

Primary GC tissues and matched non-tumor tissues were obtained from 150 GC patients undergoing radical gastrectomy at the Department of Surgery, Ruijin Hospital, School of Medicine, Shanghai Jiao Tong University. Samples were snap-frozen directly after surgery. All samples were confirmed by independent pathological examination. None of the patients received preoperative treatment. For all patients, clinicopathological information was available. Tumor classification according to the International Union Against Cancer (2009).

### RNA Isolation and Quantitative Real-time PCR (qRT-PCR)

Total RNA was extracted from cell lines and tissue samples using Trizol reagent (Invitrogen, Carlsbad, USA) according to the manufacturer’s instructions. Concentrations and purity of the RNA samples were measured by electrophoresis and spectrophotometric methods. The expression levels of miR-202-3p and U6 small nuclear RNA (RNU6B) were assayed in triplicates by the stem-loop RT-PCR method using the Hairpin-it™ miRNAs qPCR Quantitation Kit (GenePharma, Shanghai, China) with specific primers formiR-202-3p and U6 small nuclear RNA (RNU6B). Relative miRNA expression of miR-202-3p was normalized against the endogenous control, U6, using the DDCt method. The mRNA levels of Gli1 and GAPDH were measured in triplicates using the SYBR Green real time PCR (Applied Biosystems, USA) following the manufacturer’s instruction. Quantification was done using the DDCt relative quantification method with Human GAPDH as an internal control. The following primers were used: Gli1 (sense: 5′-GGA AGT CAT ACT CAC GCC TCG A-3′; antisense: 5′-CAT TGC TGA AGG CTT TAC TGC A-3′) [31] and GAPDH (sense: 5′-GGA CCT GAC CTG CCG TCT AG-3′; antisense: 5′-GTA GCC CAG GAT GCC CTT GA-3′).

### Transient Transfection of miRNA Mimics

MiR-202-3p mimics (dsRNA oligonucleotides) and negative control mimics1 (NC)(sense: 5′-UUC UCC GAA CGU GUC ACG UTT-3′, antisense: 5′-ACG UGA CAC GUU CGG AGA ATT-3′) were purchased from GenePharma (Shanghai, China). Cells were seeded into 6-well plates the day before transfection to ensure 40% cell confluence at the moment of transfection. Transfection of miRNA mimics into cells was carried out with Lipofectamine 2000™ (Invitrogen, Carlsbad, CA, USA) according the manufacturer’s procedure. The miRNA mimics were used at a final concentration of 100 nM.

### Cell Proliferation Assay

At 24 h post-transfection with miRNA mimics, cells (2×10^3^ cells/well) were seeded into 96-well plates and incubated for 72 hours. Cell proliferation was assessed in triplicates by water-soluble tetrazolium salt (WST) assay using the Cell Counting Kit-8 (Dojindo, Kumamoto, Japan) and measured following the manufacturer’s instruction.

### Soft Agar Colony Formation Assay

MiRNA mimics transfected cells were resuspended with 0.3% soft agarose (A9045, low gelling temperature, Sigma-Aldrich, USA) in RPMI 1640 containing 10% FBS and layered onto 0.4% solidified agar in RPMI 1640 containing 10% FBS in 6-well plates (1×10^3^ cells/well) at 24 h post-transfection. The plates were incubated for 2 weeks. Colonies containing at least 50 cells were counted.

### Apoptosis Analysis

One day before transfection with miRNA mimics, 1×10^6^ cells were seeded into 6-well plates. Forty-eight hours after transfection, cells were harvested and stained with AnnexinV/PI double staining kit (BD biosciences, USA) according to the manufacturer’s protocol. Apoptotic cells were assessed in triplicates and repeated three times independently by flow cytometry on a FACScan (Beckman Instruments, Fullerton, CA, USA).

### Retroviral Transfection for Stable Cell Lines

Genomic region that included the primary transcript of miR-202-3p was cloned into the EcoRI-XhoI site of the modified pMSCV-GW-RfA-PGK-EGFP retroviral vector. Negative control vectors had no insert. HEK 293T cells (1×10^6^/well) were seeded in 6-well plates the day before transfection. Ten ug of retroviral construct containing either miR-202-3p or no insert, 2 ug of gag/pol and 2 ug of VSVG were co-transfected into HEK 293T cells using Lipofectamine™ 2000 reagent and Opti-MEM I reduced serum medium in each plate. Viruses were harvested at 48 h and 72 h post-transfection by viral collection medium (RPMI-1640 with 10% heat-inactivated FBS +1% Glutamine +20 mM Hepes). Infections of MKN-28 cells were carried out in the presence of 8 µg/mL of polybrene in each well of a 6-well plate. Cells were spin infected at 1500 rpm for 30 min at room temperature. Virus-containing supernatant was removed after 2 h. Positive cells were selected for GFP expression by FACS-sorting and named RV-miR-202-3p and RV-miR-control respectively. MiR-202-3p expression was confirmed by qRT-PCR.

### Tumor Growth in Nude Mice

Male BALB/c nu/nu mice, at age of six weeks (Institute of Zoology Chinese Academy of Sciences), were housed at a specific pathogen-free environment in the Animal Laboratory Unit, School of Medicine, Shanghai Jiao Tong University, China. Mice received humane care and the study protocols were carried out according to a protocol approved by the Institutional Animal Care and Use Committee (IACUC) at Shanghai Jiao Tong University, Shanghai, China. Cells (100 µl, 2×10^6^ cells) from stable transfected lines RV-miR-202-3p or RV-miR-control were collected and inoculated subcutaneously into mice. Six mice were used for each group. Mice were checked weekly, and tumor nodules were measured with a caliper. Tumor volume (V) was estimated by using the equation V = 4/3π ×L/2×(W/2)^2^, where L is the mid-axis length, and W is the mid-axis width.The tumor cells were allowed to growth 5-weeks and tumor growth curves and inhibiting rates were calculated. After the mice were sacrificed, all tumor grafts were excised, weighed, harvested, fixed, and embedded. Each experiment was performed twice.

### TUNEL Analysis

The terminal nucleotidyl transferase-mediated nick end labeling assay (TUNEL) was performed following the manufacturer’s instructions of the DeadEnd™ Colorimetric TUNEL System kit (Promega, USA). Tissues were fixed in 10% neutralized formalin and embedded in paraffin blocks. Sections (4 µm) were then prepared for examination. After deparaffinization, the sections were treated with 20 g/ml proteinase K for 10 min, with 0.3% H_2_O_2_ in methanol for 10 min and 0.1% Triton X-100 in 0.1% sodium citrate for 2 min on ice. Then the sections were incubated with TUNEL reaction mixture for 60 min at 37°C. Further incubation with peroxidase-conjugated antibody was performed for 30 min at 37°C. The sections were stained with diaminobenzidine solution for 10 min at room temperature and then counterstained with hematoxylin.

### Immunohistochemistry

Sections (6 mm thick) of formalin-fixed, paraffin-embedded tumor specimenswere deparaffinized in xylene and rehydrated in graded alcohol. Endogenous peroxidase was blocked using 3% H_2_O_2_ for 10 min. Following antigen retrieval in citrate buffer (pH 6.0), the tissue sections were incubated with normal goat serum to block nonspecific antibody binding (20 min at room temperature). The sections were then incubated with Gli1 antibodies (1∶50, sc-20687, Santa Cruz Biotechnology Inc., Santa Cruz, CA) at 37°C in humidchambers for 2 h. After washing with PBS three times, the sections were incubated with peroxidase-conjugated anti-rabbit IgG for 1 h at room temperature. Next, the slides were rinsed with PBS and incubated for 5 min with DAB substrate for less than 30 min. Haematoxylin was used for counterstaining.

### Construction of Plasmids and Luciferase Activity Assay

A 203-bp full length of the wild-type (WT) Gli1-3′UTR or mutant Gli1-3′UTR (mut) containing the putative miR-202-3p binding site was synthesized (Sangon, Shanghai, China). After digestion by SpeI and HindIII, the fragments of wild-type and mutant Gli1-3′UTR were cloned into the SpeI and HindIII sites of pMIR-Report Luciferase vector (Applied Biosystems) and named pMIR/Gli1 and pMIR/Gli1/mut, respectively. DNA sequencing was used to verify the constructs.

HEK-293T cells were seeded in 24-well plates 24 h prior to assay performance. In each well, 100 ng pMIR/Gli1 or pMIR/Gli1/mut, 2 ng pRL-TK (Promega, Madison, WI, USA) containing Renilla luciferase and 100 nM miRNA mimics were cotransfected using Lipofectamine™ 2000 reagent and Opti-MEMI reduced serum medium. Relative luciferase activity was calculated 48 h after cotransfection using Dual-Glo Luciferase assay (Promega, USA) according to the manufacturer’s procedure. Firefly luciferase activity was normalized to Renilla luciferase activity.

### Western Blot Analysis

Cells and tumor were lysed using M-PER reagents and Halt Protease Inhibitor Cocktail kits (Pierce, USA). The protein concentration of the cell lysates was quantified using a BCA Protein Assay Kit (Pierce, USA). Protein were separated by SDS polyacrylamide gel electrophoresis and blotted onto 0.22-µm polyvinylidene difluoride membranes (Millipore, MA, USA). The following specific antibodies were used: Gli1 (1∶1000, Cell Signaling Technology), γ-catenin (1∶2000, BD Biosciences, USA), BCL-2 (1∶2000, EPITMICS, USA), and GAPDH (1∶20000, Abcam, UK). Protein levels were normalized to total GAPDH.

### Construction of Gli1 Expression Plasmid and Transfection

By amplifying cDNA of MKN-28 using the following primers: sense (5′- AGT TGA ACA TGG GAT ATC AT-3′) and antisense (5′- ATG CGG CCG CTT AGG CAC TA-3′), full-length Gli1 was obtained, digested by EcoR V and Not I and cloned into pcDNA3.1 vector (Invitrogen) and named pcDNA3.1-Gli1. DNA sequencing was used to verify the constructs.

Cells were seeded into 6-well plates the day before transfection to ensure 80–90% cell confluence at the moment of transfection. In each well, cells were transfected with 250 pmol of miR-202-3p mimics or control, together with 4 ug of pcDNA3.1-Gli1 or the pcDNA3.1 empty vector, by using Lipofectamine 2000. WST assay was performed at 24 h post-transfection, while apoptosis analysis was carried out at 48 h post-transfection.

### Statistical Analysis

Continuous variables were compared using the Student’s *t* test for normally distributed variables and Wilcoxon rank-sum test for nonnormally distributed variables. The relationship between the miR-202-3p expression levels and clinicopathologic parameters was analyzed using the Pearson Chi-square test. All values are presented as means ± SDs. All statistical analyses were performed using PASW Statistics 18.0 software (IBM, USA). A two-tailed value of *P*<0.05 was considered statistically significant.

## Results

### The expression of miR-202-3p is Decreased in GC and Correlates with Clinicopathologic Parameters

**Figure 1:**
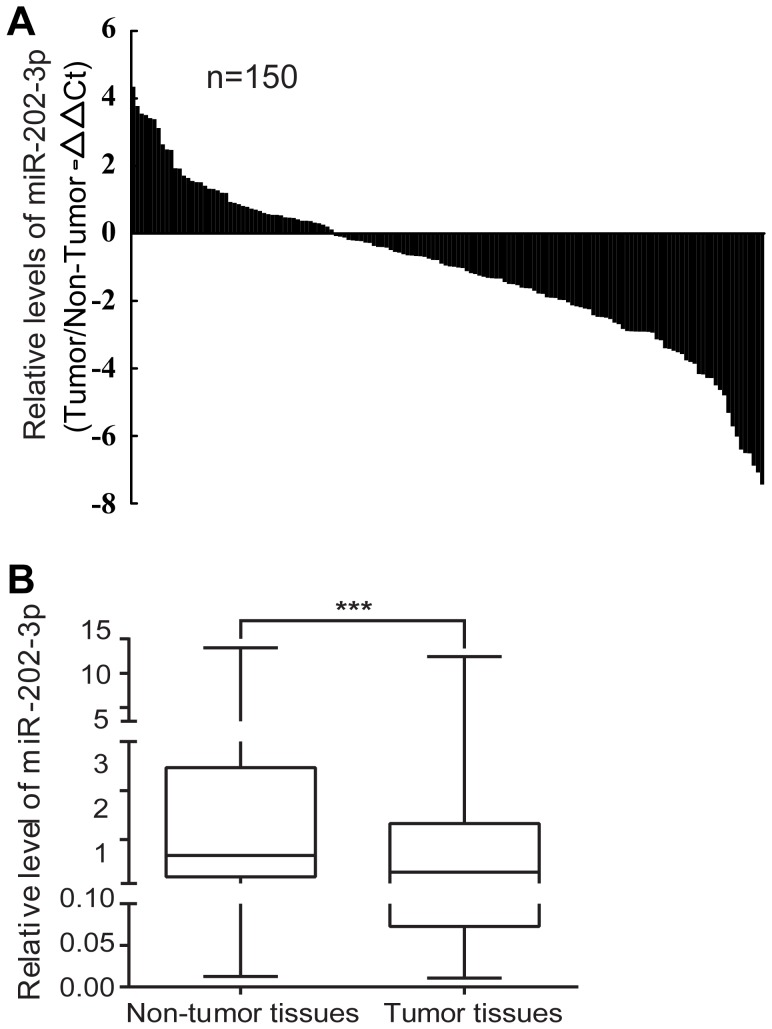
MiR-202-3p is downregulated in GC. (A) Relative expression of miR-202-3p in 150 GC patient tissues compared with its adjacent non-tumor tissues. qRT-PCR results are shown as −ΔΔCT values. (B) The boxes represent the 25th through 75th percentiles. The horizontal lines represent the medians. The whiskers represent the 10th and 90th percentiles. ***, *P*<0.001.

Expressions of miR-202-3p were examined by qRT-PCR in tumor tissues and the matched non-tumor tissues from 150 GC patients (Fig. 1A). The results show that expression of miR-202-3p was significantly downregulated in tumor tissues compared with matched non-tumor tissues in 68% (102/150) of the GC patients (p<0.001; Fig. 1A, B).

To further elucidate the correlation between expression level of miR-202-3p and clinicopathologic factors in human GC, the 150 cases were further analyzed (Table 1). Based on relative miR-202-3p expression, the 150 cases were stratified into 3 groups: miR-202-3p low expression (tumor/non-tumor ratio<0.66, n = 85), miR-202-3p moderate expression (tumor/non-tumor ratio 0.66-1.5, n = 34) and miR-202-3p high expression (tumor/non-tumor ratio >1.5, n = 31). The miR-202-3p expression levels were negatively correlated to tumor size and positively correlated to age in these patients, with the miR-202-3p low expression group exhibited significantly larger tumor size (p = 0.013) and elder age compared with moderate or high expression groups (p = 0.028). However, miR-202-3p expression levels did not show any relationship with gender, differentiation, tumor location, tumor local invasion, lymph node metastasis or TNM stage.

**Table 1:** Relationship between miR-202-3p expression level and clinicopathologic parameters in 150 GC cases.

Clinicopathologic parameters	miR-202-3p expression			p-value
	Low (n = 85)	Middle(n = 34)	High (n = 31)	
Age (years)				
<60	37	21	9	0.028*
≥60	48	13	22	
Gender				
Male	60	23	23	0.845
Female	35	11	8	
Tumor size(cm)				
≥5	61	21	13	0.013*
<5	24	13	18	
Differentiation				
High, middle	21	10	13	0.197
Low	64	24	18	
Location				
Distal third	45	20	17	0.844
Middle third,proximal third	40	14	14	
Local invasion				
T1, T2	24	11	7	0.679
T3, T4	61	23	24	
Lymph node metastasis				
No	21	8	6	0.833
Yes	64	26	25	
TNM stage				
I, II	34	19	12	0.242
III, IV	51	15	19	

* p-value<0.05 was considered statistically significant.

### Overexpression of miR-202-3p Inhibits GC Cell Proliferation

Given that miR-202-3p is significantly decreased in GC, it may function as a tumor suppressor. Therefore, we examined whether overexpression of miR-202-3p in GC cells affected cell growth. MKN-28 and BGC-823 cell lines, whose expression of miR-202-3p were the lowest in the eight tested GC cell lines (Fig. 1C), were chosen for the subsequent expreiments. Synthetic miR-202-3p mimics and negative control mimic molecules (NC) were transfected into MKN-28 and BGC-823 cells respectively. The ectopic expression of miR-202-3p in cells was confirmed by qRT-PCR.

**Figure 2:**
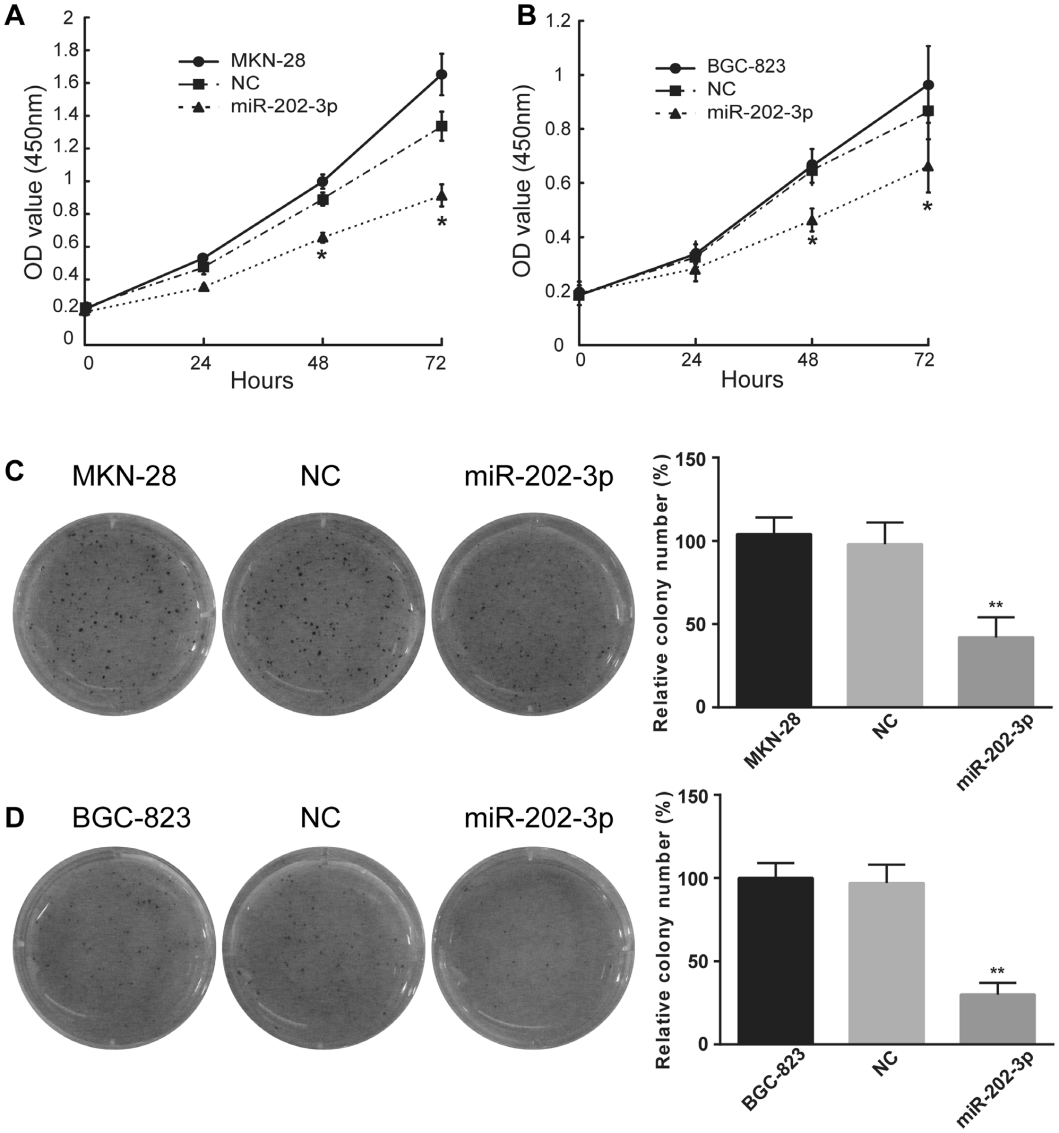
Overexpression of miR-202-3p promotes the proliferation of GC cells *in vitro*. Cell proliferation was evaluated by the WST assay in MKN-28 cells (A) and BGC-823 cells (B). Cells were transfected with miR-202-3p mimics or NC at 24 h post-transfection were subjected to WST assay along with parental cells. The results are means of three independent experiments ± S.D. *, *P*<0.05. The effect of miR-202-3p on cell proliferation was assessed by the colony formation assay in MKN-28 cells (C) and BGC-823 cells (D). Cells transfected with 100 nM miR-202-3pmimics or control were seeded onto 6-well plates along with parental cells. The number of colonies was counted on the 14th day after seeding. Representative photographs of colonies are shown in left panels. Relevant quantification of the results is shown in the bar graphs (right panels). The results are means of three independent experiments ± S.D. *, *P*<0.05.

The WST cell-growth assay showed significant cell growth inhibitions in MKN-28 cells transfected with miR-202-3p (Fig. 2.A). Similar results were observed in BGC-823 cells (Fig. 2.B). To further characterize the effect miR-202-3p on cell growth, we performed soft agar colony formation assay in transfected cells. We found that the number of colonies from MKN-28 cells transfected with the miR-202-3p mimics was nearly half of that from the control and parental group (Fig. 2C). Similar results were observed in BGC-823 cells (Fig. 2.D). These results demonstrate that miR-202-3p can suppress the cell proliferation of GC cells *in vitro*.

### Overexpression of miR-202-3p Induces GC Cells Apoptosis

**Figure 3:**
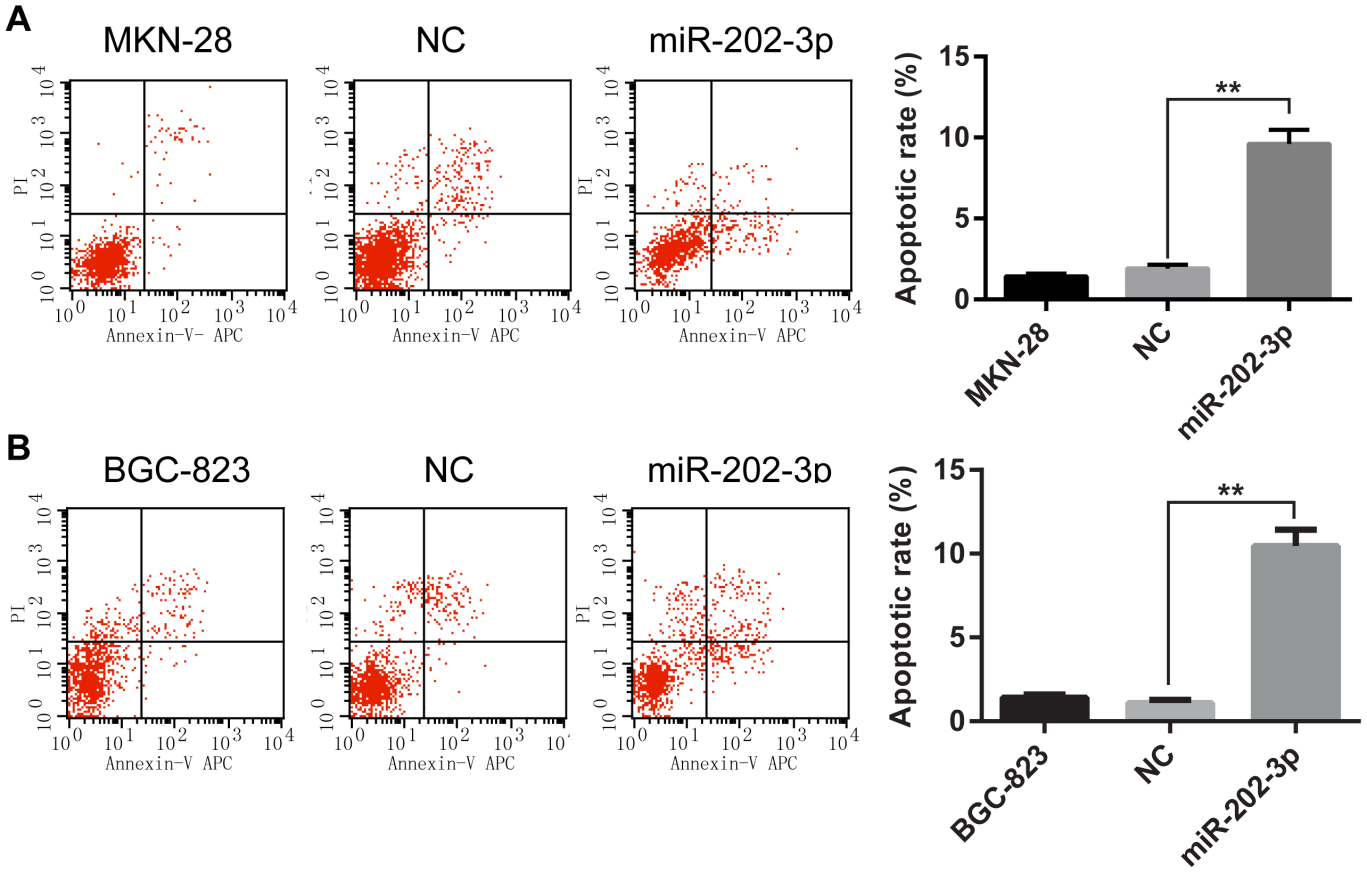
Effect of miR-202-3p on apoptosis in GC cells. (A) Representative histograms depicting apoptosis of MKN-28 cells transiently transfected with 100 nM miR-202-3p mimics or NC and parental cells (left panels). The percentage of apoptotic cells of three independent experiments ± S.D. are shown in the bar graphs (right panels). (B) Representative histograms depicting apoptosis of BGC-823 cells transiently transfected with 100 nM miR-202-3p mimics or NC and parental cells (left panels). The percentage of apoptotic cells of three independent experiments ± S.D. are shown in the bar graphs (right panels).

Since growth inhibition might due to blocked cell cycle progression or increased apoptosis, we next preformed cell division cycle assay and apoptosis by flow cytometry. Our data indicated that the apoptotic rates of both MKN-28 and BGC-823 cells transfected with miR-202-3p mimics were significantly upregulated (Fig. 3A, B). However, there was no significant difference in cell cycle between differently treated groups (data not show). These results indicated that overexpression of miR-202-3p induces apoptosis in GC cells may contributes to the growth inhibitory properties of miR-202-3p.

### Overexpression of miR-202-3p Inhibits Tumorigenicity and Increases Apoptosis *in vivo*

**Figure 4:**
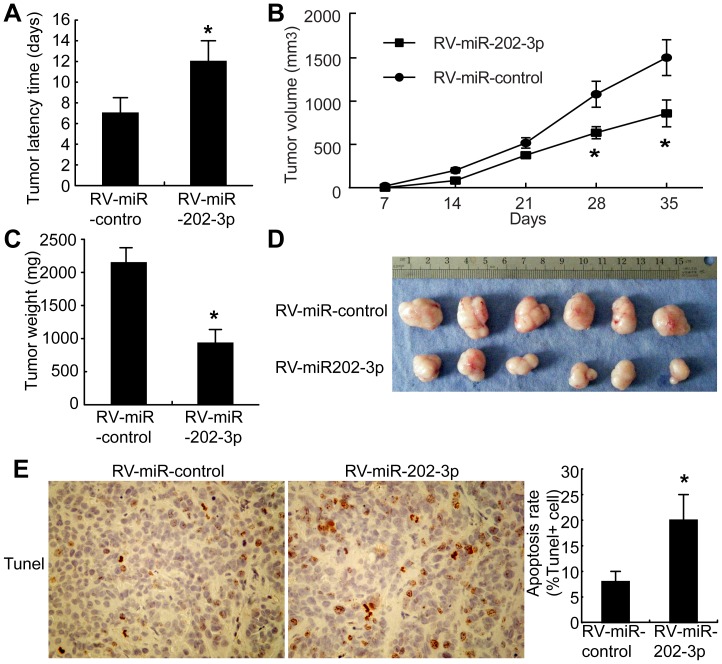
Overexpression of miR-202-3p inhibits tumorigenicity and increases apoptosis *in vivo.* (A) The tumor latency was the number of days to the onset of palpable tumor and the values are given as mean ± S.D. *, *P*<0.05. (B) Tumor growth curves were measured after injection. Tumor diameters were measured every 7 days. (C) Tumor weight; the values are given as mean± S.D. *, *P*<0.05. (D) The photograph shows representative features of tumor xenografts 35 days after inoculation. (E) Representative images of TUNEL assay of tumor xenografts of mice (left panels). The percentage of apoptotic cells was counted (right panels). The results are means of three independent experiments ± S.D. *, *P*<0.05.

Given that miR-202-3p inhibits GC cells growth and induces apoptosis *in vitro*, we next examined whether miR-202-3p could suppress tumor growth and induce apoptosis *in vivo*. Cells of MKN-28, RV-miR-202-3p (MKN-28 cells with retrovirus-mediated miR-202-3p stable expression) or RV-miR-control (MKN-28 cells with the empty vector) were obtained as described in Material and Methods. Cells were injected subcutaneously into nude mice. Tumor formation was monitored. The tumor latency time showed a significant difference between the mice injected with RV-miR-NC cells and RV-miR-202-3p cells (Fig. 4A). It is of considerable interest that tumors derived from RV-miR-202-3p cells grew substantially more slowly than the RV-miR-control group throughout tumor growth (Fig. 4B). The average weight of tumors resulting from RV-miR-202-3p was significantly less than tumors derived from RV-miR-control (Fig. 4C, D).

**Figure 5:**
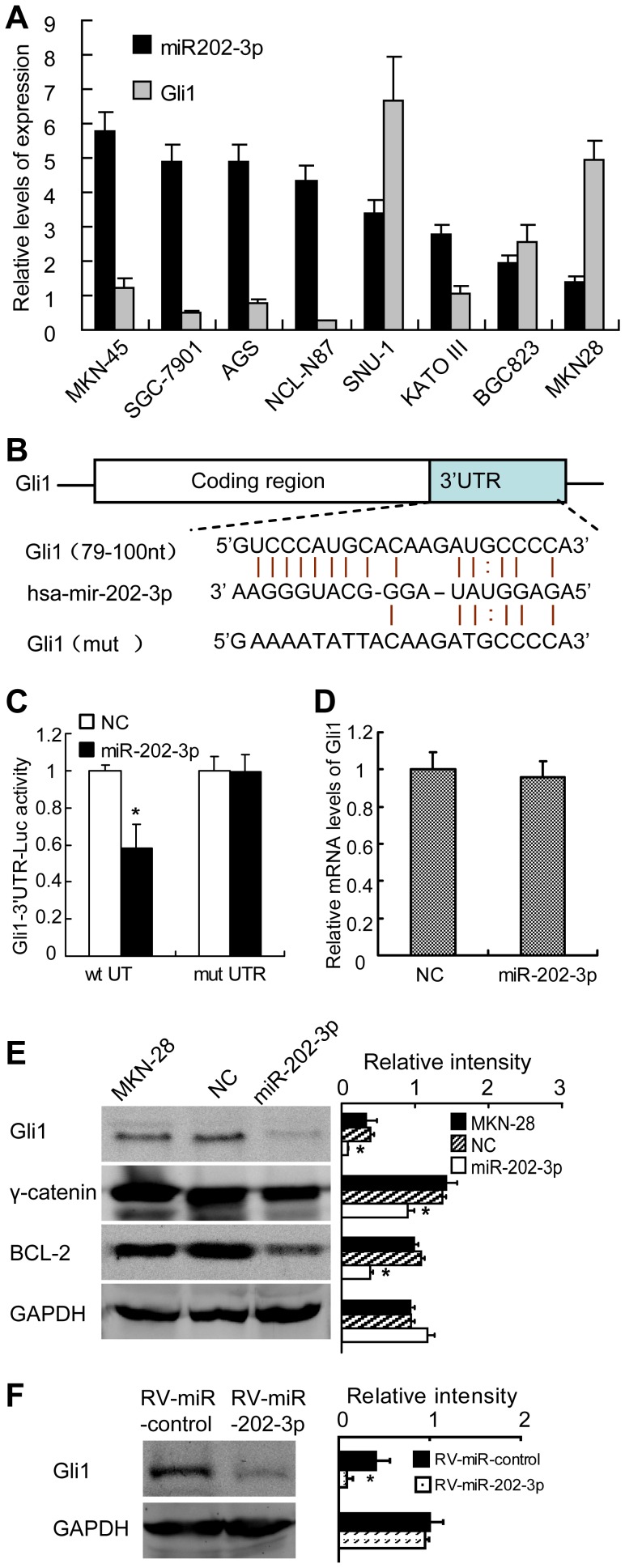
MiR-202-3p targets Gli1 and inhibits γ–catenin and BCL-2 expression. (A) Relative expression of miR-202-3p and Gli1 in eight GC cell lines was carried out by qRT-PCR. Results are means of three independent experiments ± S.D. (B) Sequence of the Gli1 3′UTR showing the miR-202-3p binding site and mutation of the Gli1 3′UTR binding site to create Gli1-mut. (C) miR-202-3p mimics down-regulated luciferase activities controlled by wild-type Gli1 3′UTR (*P*<0.01), but did not affect luciferase activity controlled by mutant Gli1 3′UTR. The results are means of three independentexperiments ± S.D. (D) Gli1 mRNA in MKN-28 cells was analyzed by qRT-PCR at 48 h post-transfection with miR-202-3p mimics and NC. (E) Representative Western blotting images of indicated protein in MKN-28 cells (left panels), with relevant quantification (right panel). The results are means of three independent experiments ± S.D. *, *P*<0.05. (F) Gli1 protein was detected in RV-miR-control or RV-miR-202-3p cells from subcutaneous tumor in nude mice (left panels), with relevant quantification (right panel). The results are means of three independent experiments ± S.D. *, *P*<0.05.

Additionally, TUNEL assays of tumor tissues were performed. As shown in Fig. 5E, the RV-miR-202-3p cells showed a more tumor cell positive staining and a significantly higher apoptotic index than the RV-miR-control cells (*P*<0.01). These results suggest that miR-202-3p inhibition of tumorigenicity is attributed to increased apoptosis *in vivo*.

### MiR-202-3p Directly Target Gli1

To understand the mechanism underlying the tumor-inhibiting properties of miR-202-3p in GC, we searched for its putative target genes with potential pro-oncogenic functions using online search tools (RNAhybrid and miRanda algorithms), and the genes predicted by both of the bioinformatic tools were chosen as the candidate target genes of miR-202-3p. Among the hundreds of candidate genes predicted, the transcription activator Gli1 is of particular interest (Figure S1). The putative secondary structure of RNA hybrid for human miR-202-3p and Gli1 is shown in Figure S2. Gli1 has been reported highly expressed in GC [32], [33]. It suggested that decreasing expression of Gli1 resulted in GC cell lines growth inhibition and apoptosis [33].

A further hint about the potential role of miR-202-3p in the regulation of Gli1 expression came from the analysis of eight different gastric cancer cell lines (MKN-45, SGC-7901, AGS, NCI-N87, SNU-1, KATO III, BGC-823 and MKN-28). Statistical analysis showed a significant inverted patterns expression between miR-202-3p and Gli1 ((r = −0.345, *P*<0.001, Fig. 5A).

To assess the clinical relevance of these findings, we examined the correlation between the expressions of Gli1 with miR-202-3p expression in GC tissues. We found that Gli1 and miR-202-3p exhibit inverse expression pattern in GC tissues (Table 2). These results support the premise that downregulation of miR-202-3p increases the level of Gli1 gene in gastric cancer.

**Table 2:** Gli1 and miR-202-3p exhibit inverse expression pattern in GC samples.

	miR-202-3p expression			p-value
	Low(n = 85)	Middle(n = 34)	High(n = 31)	
Gli1(IHC)				
Positive	71 (83.5%)	24 (70.6%)	19 (61.3%)	0.032*
Negative	14 (16.5%)	10 (29.4)	12 (38.7%)	

* p-value<0.05 was considered statistically significant.

Luciferase reporter assays were performed to verify a direct interaction between miR-202-3p and the 3′UTR of Gli1. Luciferase reporters were constructed containing either a wild-type Gli1 3′UTR sequence including the miR-202-3p binding site (pMIR/Gli1), or a mutated Gli1 3′UTR (pMIR/Gli1/mut) (Fig. 5B). The pMIR/Gli1 and pMIR/Gli1/mut luciferase reporter constructs were transfected into HEK-293T cells, along with miR-202-3p or NC mimics. The relative luciferase activity of the pMIR/Gli1 reporter was markedly suppressed compared with that of pMIR/Gli1/mut in a miR-202-3p-dependent manner (Fig. 5C). This result strongly indicates that 3′UTR of Gli1 carries the direct binding sites of miR-202-3p.

To determine at mRNA level or protein level miR-202-3p down-regulated Gli1, we detected Gli1 expression by qRT-PCR and Western blot. As shown in Fig. 5E, the Gli1 protein level was decreased in miR-202-3p mimics-transfected MKN-28 cells compared with NC mimics-transfected and parental MKN-28 cells. Meanwhile, there was not any effect of miR-202-3p on the Gli1 mRNA level (Fig. 5D). These results strongly suggest that miR-202-3p negatively regulates Gli1 expression at the translational level.

Among the genes reported to inhibit the apoptosis of GC, γ-catenin (Plakoglobin) and BCL-2 are direct transcriptional targets of Gli1 [34], [35]. As shown in Fig. 5E, the protein levels of γ-catenin and BCL-2 were markedly reduced in MKN-28 cells transfected with miR-202-3p mimics.

In addition, we examined expression of the Gli1 protein in subcutaneous tumors derived from RV-miR-control and RV-miR-202-3p in nude mice by western blot analysis. The Gli1 protein level in tumors from RV-miR-202-3p demonstrated downregulation compared with that in RV-miR-control (Fig. 5F). These results strongly suggest that miR-202-3p negatively regulates Gli1 expression post-transcriptionally.

### Overexpression of Gli1 Rescues Effect of miR-202-3p in GC Cells

**Figure 6:**
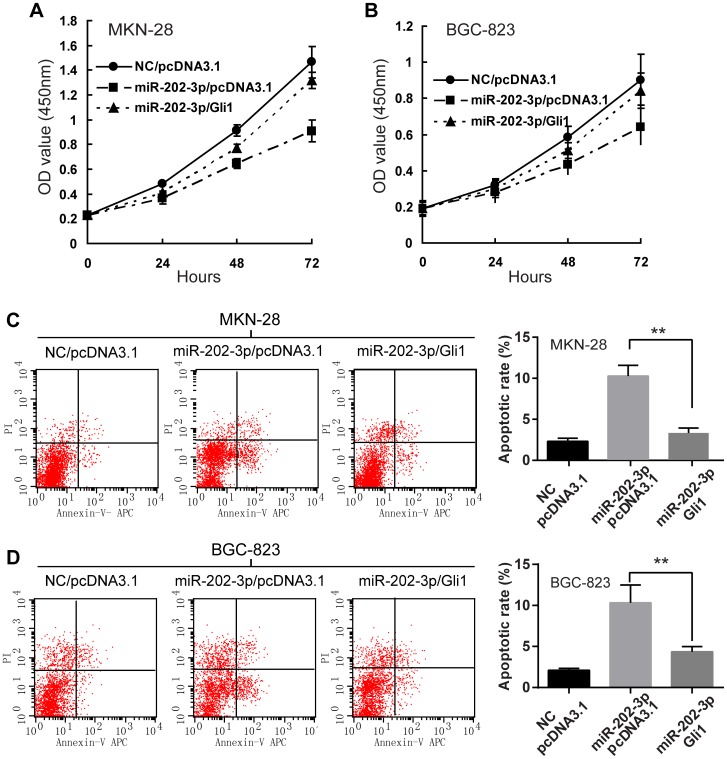
Overexpression of Gli1 rescues effect of miR-202-3p in GC cells. Upon transfected with Gli1 expression vector (pcDNA3.1-Gli1), MKN-28 cells (A) and BGC-823 cells (B) were partially rescued from miR-202-3p growth inhibitory properties. Cells were transfected with miR-202-3p mimics or control, together with pcDNA3.1-Gli1or pcDNA3.1 empty vector (pcDNA3.1) at 24 h post-transfection were subjected to WST assay. The results are means of three independent experiments ± S.D. *, *P*<0.05. Representative histograms depicting apoptosis MKN-28 cells (C) and BGC-823 cells (D) transfected with miR-202-3p mimics or control, together with pcDNA3.1- Gli1) or pcDNA3.1 by flow cytometry (left panels). The percentage of apoptotic cells of three independent experiments ± S.D. are shown in the bar graphs (right panels).

Since miR-202-3p downregulated Gli1 to inhibit cell proliferation and induce apoptosis, it is reasoned that overexpression of Gli1 could reverse this phenomenon at least in part. Indeed, when Gli1 overexpressing plasmid (pcDNA3.1-Gli1) was introduced into MKN-28 or BGC-823 cells transiently transfected with miR-202-3p mimics, the inhibitory effect of miR-202-3p on cell growth was partially reversed, which was observed by WST cell growth assay (Fig. 6A,B). Moreover, progression toward apoptosis was also hindered when Gli1 was overexpressed in MKN-28 or BGC-823 cells transiently transfected with miR-202-3p mimics (Fig. 6C,D).These data provide evidence that growth inhibition and cell apoptosis induced by miR-202-3p is at least partially related to its effect on Gli1 expression.

## Discussion

Accumulating evidences have demonstrated dysregulation of specific miRNA in GC [4], [13], [36]. MiR-202-3p, located in 10q26, has been reported to be disregulated in breast cancer [23], [24], colorectal cancer [26] and follicular lymphoma [27]. Petrocca et al. found that miR-202 was down-regulated in chronic gastritis [28]. However, the role of miR-202-3p in GC development and progression is still unkown. In this study, we find that miR-202-3p expression of 68% gastric carcinoma samples was significantly lower than that of matched normal tissues, suggesting that reduced miR-202-3p expression is a frequent event in GC. Importantly, miR-202-3p levels are negatively correlated with tumor size of GC in patients. These results suggest the inhibitory role of miR-202-3p in the development and progression of GC.

Given miR-202 was down-regulated in gastric cancer tissues, we speculated that overexpression of miR-202 might suppress the malignant phenotypes of gastric cancer cells. In the present study, we show that restoration of miR-202-3p in MKN-28 and BGC-823 cells significantly inhibits cell proliferation and induces apoptosis both *in vitro* and *in vivo*. These results strongly demonstrated that the decreased miR-202-3p expression in GC should be a factor contributing to the development rather than being affected as a consequence of GC. Therefore, the significant inhibition of tumor xenografts in nude mice imply that therapeutic strategies of introducing miR-202-3p into cancer cells might be useful for retarding the process of tumorigenesis.

MiRNAs can regulate gene expression through down-regulated translation of target mRNA or up-regulated degradation of target mRNAs [3], [37]. Based on bioinformatic algorithms, we identify Gli1 as a possible direct target gene of miR-202-3p. Gli1, a member of Gli family, was originally identified as an amplified gene in a malignant glioma [38]. It is a strong positive activator of downstream target genes and is itself a transcriptional target of Shh signaling [39], and it can also be upregulated by RAS/PKC [40], TGFβ [41] and PI3K signaling [42], and downregulated by PKA [42] and p53 signaling [43]. Gli1 expression in epithelial cells can induce cell transformation characterized by increased proliferation and anchorage-independent proliferation [35], [44]. Increasing number of studies show that Gli1 is overexpressed in various cancers including gastric cancer [32], [33], [45], and the expression levels of Gli1 are positively correlated with tumor differentiation [33]. Decreasing expression of Gli1 by cyclopamine or siRNA resulted in GC cell growth inhibition and apoptosis [33]. We further validate Gli1 as a direct target of miR-202-3p, we found that miR-202-3p bounding with incomplete complementarity to Gli1 3′UTR, and overexpression of miR-202-3p down-regulates Gli1 at the protein level. Moreover, statistical analysis showed a significant inverted patterns expression between miR-202-3p and Gli1 in GC tissues and cell lines. γ-catenin (Plakoglobin) and BCL-2, direct transcriptional targets of Gli1, are reported to inhibit the apoptosis of GC. The expression of Gli1 downstream target genes γ-catenin (Plakoglobin) and BCL-2 [34], [35] are markedly reduced when miR-202-3p is overexpressed in GC cells. This suggests that the growth inhibition of GC cells induced by miR-202-3p might partially related to its suppression on γ-catenin and BCL-2 expression, which were in turn through direct interaction with Gli1. Importantly, overexpression of Gli1 rescues the cellular growth inhibition and cell apoptosis induced by miR-202-3p, further demonstrating that Gli1 is a direct target of miR-202-3p and suggesting an essential role for Gli1 as a mediator of the biological effects of miR-202-3p in GC.

**Figure 7:**
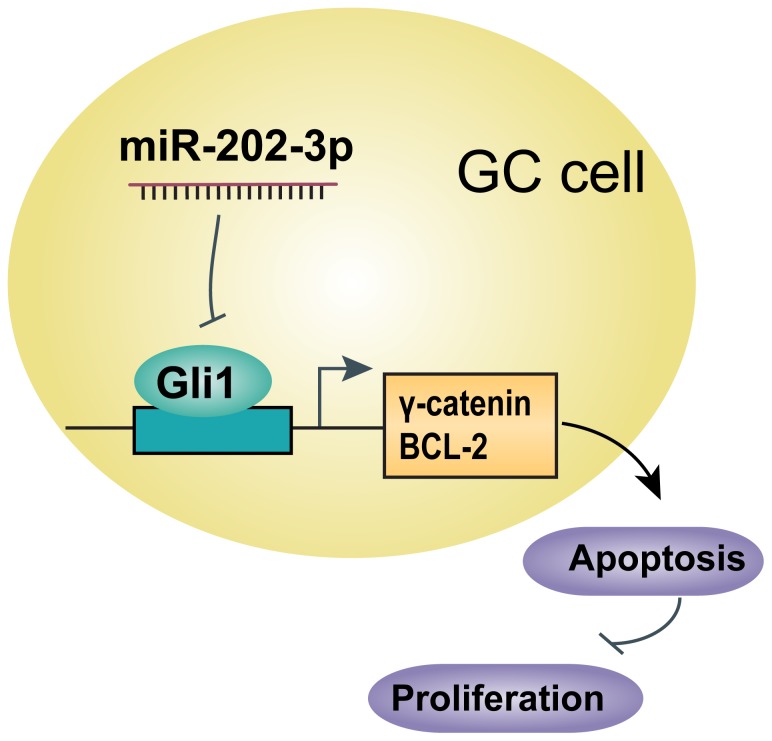
Diagram of interrelationship between mIR- 202-3p and proliferation in GC cells. Expression of Gli1 is inhibited by interaction with miR-202-3p directly. Gli1 can upregulate the expression of γ-catenin and BCL-2, leading to induction of proliferation and reduction of apoptosis in GC cells.

In summary, miR-202-3p is frequently decreased in human gastric cancer. Restoration of miR-202-3p inhibits GC proliferation at least partly through inducing cell apoptosis by direct interaction with Gli1 (Fig. 7). Such roles of miR-202-3p in GC suggest its potential as a therapeutic microRNA for gastric cancer treatment, which is worth of further research.

## Supporting Information

Figure S1 **Schematic diagram of predicted target site of miR202-3p (has-miR-202) in the Gli1 3′-UTR using miRanda and RNAhybrid tools in miRNAMap website.**
(PNG)

Figure S2 **Schematic diagram of secondary structure of hybrid of miR202-3p (green) and Gli1 3′-UTR(red) using RNAhybrid 2.2.**
(PNG)
