# Supplementary figures and images for: Decrease of miR-202-3p Expression, a Novel Tumor Suppressor, in Gastric Cancer
Source: PLoS One. 2013 Jul 25;8(7):e69756. doi: 10.1371/journal.pone.0069756 (PMC3723650; doi:10.1371/journal.pone.0069756)

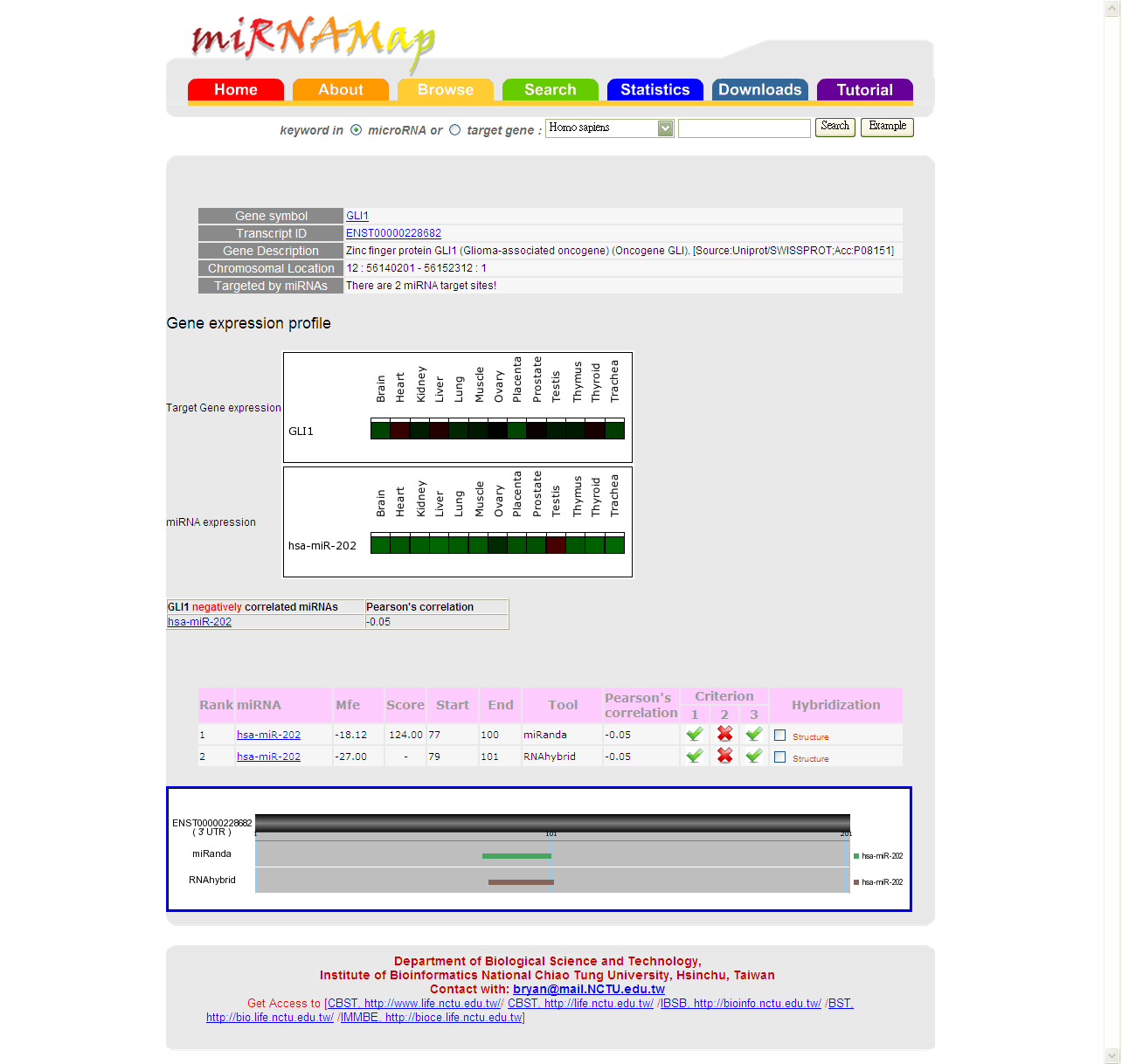

Supplement: Figure S1 — Schematic diagram of predicted target site of miR202-3p (has-miR-202) in the Gli1 3′-UTR using miRanda and RNAhybrid tools in miRNAMap website. (PNG) [file pone.0069756.s001.png]

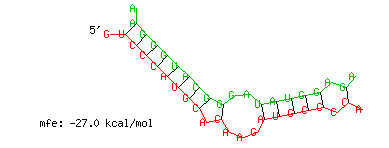

Supplement: Figure S2 — Schematic diagram of secondary structure of hybrid of miR202-3p (green) and Gli1 3′-UTR(red) using RNAhybrid 2.2. (PNG) [file pone.0069756.s002.png]
